# Bis(tetra­ethyl­ammonium) dodeca­cyanododecaborate(2−)

**DOI:** 10.1107/S2056989026008480

**Published:** 2026-08-28

**Authors:** Marcus A. Juhasz, Ashlyn A. Kamin

**Affiliations:** a Department of Chemistry Whitman College, 345 Boyer Ave Walla Walla WA USA 99362; University of Massachusetts Dartmouth, USA

**Keywords:** crystal structure, boranes, borates, boron clusters, microwave synthesis, cyanation

## Abstract

The title compound, 2C_8_H_20_N^+^·B_12_C_12_N_12_^2−^, was prepared using microwave-promoted cross-coupling and crystallized from an aceto­nitrile-water solution. The structure was determined at 101 K, indicating the title compound crystallizes in the trigonal space group *P*3 and features a B_12_(CN)_12_^2−^ subunit with nearly icosa­hedral symmetry. The anionic subunit interacts with the Et_4_N^+^ cation in the structure by multiple weak C*sp*^3^–H⋯N hydrogen bonds between CN groups on the anion and methyl and methyl­ene H atoms on the cation.

## Abstract 2

Bis(tetra­ethyl­ammonium) dodeca­cyano­dodeca­borate(2−) crystallizes in the trigonal space group *P*3. The B_12_(CN)_12_^2–^ subunit has nearly icosa­hedral symmetry and interacts with the Et_4_N^+^ cation in the structure by multiple weak C*sp*^3^–H⋯N hydrogen bonds between CN groups on the anion and methyl or methyl­ene H atoms on the cation.

## 1. Chemical context

Clusters based on an icosa­hedral framework of boron atoms, such as the borane anion *closo*-B_12_H_12_^2–^ (Pitochelli & Hawthorne, 1960 ▸), and the related boron and carbon-based carborane clusters *closo*-CB_11_H_12_^−^ (Knoth, 1967 ▸) and *ortho*-C_2_B_10_H_12_ (Heying *et al.*, 1963 ▸) were first reported over 60 years ago. In contrast to many other boron hydride compounds, which are often reactive under ambient conditions, *e.g*., the simplest isolable borane, diborane (B_2_H_6_), is a pyrophoric gas, icosa­hedral boranes and carboranes are highly stable solids. *closo*-B_12_H_12_^2–^, which has the same core skeleton of twelve borons as the title compound, has been described as ‘the most stable mol­ecule in all of chemistry’ (Grimes, 2004 ▸), with its Cs^+^ salts exhibiting thermal stability under vacuum at temperatures above 800°C (Muetterties *et al.*, 1964 ▸).

Anionic boron clusters within the icosa­hedral family (*e.g. closo*-CB_11_H_12_^−^ and *closo*-B_12_H_12_^2–^) exhibit delocalization of charge within the core of twelve heavy atoms. This property contributes to their exceptional stability and makes these species useful as weakly-coordinating anions (WCAs) for applications where a tightly-bound counter-anion would attenuate the activity of a key cationic species, for example, a metal center in a catalyst. The *closo*-CB_11_H_12_^−^ and *closo*-B_12_H_12_^2–^ anions have also been suggested as promising electrolytes for batteries (Carter *et al.*, 2014 ▸; Giri *et al.*, 2014 ▸; Fisher *et al.*, 2019 ▸; Guzik *et al.*, 2019 ▸; Ceppetelli *et al.*, 2025 ▸; Akrouchi *et al.*, 2026 ▸). The replacement of hydrogens on parent boron cluster anions with sterically-hindering and/or electron-withdrawing groups (EWGs) is an established approach for improving their performance as WCAs (Strauss, 1993 ▸; Reed, 1998 ▸) and derivatization of boron anions has been investigated as a strategy for tuning their properties for charge storage applications (Bukovsky *et al.*, 2017 ▸; Ready *et al.*, 2024 ▸). Halogenation is one of the most common synthetic modifications to *closo*-CB_11_H_12_^−^ and *closo*-B_12_H_12_^2–^, and a variety of methods for halogenation of these boron clusters have been reported (Douvris & Michl, 2013 ▸; Sivaev *et al.*, 2002 ▸).

Cyano (CN) groups, one of the strongest known electron-withdrawing substituents, have been investigated as well. Initial attempts to cyanate *closo*-B_12_H_12_^2–^ by UV irradiation of perchlorinated and perbrominated precursors in the presence of KCN yielded a maximum of 9 CN groups per cluster (Trofimenko & Cripps, 1965 ▸; Trofimenko, 1966 ▸). Nearly 50 years later, computational studies predicted that the completely cyanated cluster, *closo*-B_12_(CN)_12_^2–^ would be a promising electrolyte for batteries (Zhao *et al.*, 2016 ▸). Subsequently, an updated UV-irradiation synthesis was finally achieved many years later, but the target *closo*-B_12_(CN)_12_^2–^, present in *ca*. 10–15% abundance, was not isolated from the mixture of products (Mayer *et al.*, 2020 ▸). A subsequent preparation of *closo*-B_12_(CN)_12_^2–^ by microwave-promoted cross-coupling and isolation of this anion as a tetra­ethyl­ammonium ion salt in 35% yield was reported (Kamin & Juhasz, 2020 ▸).
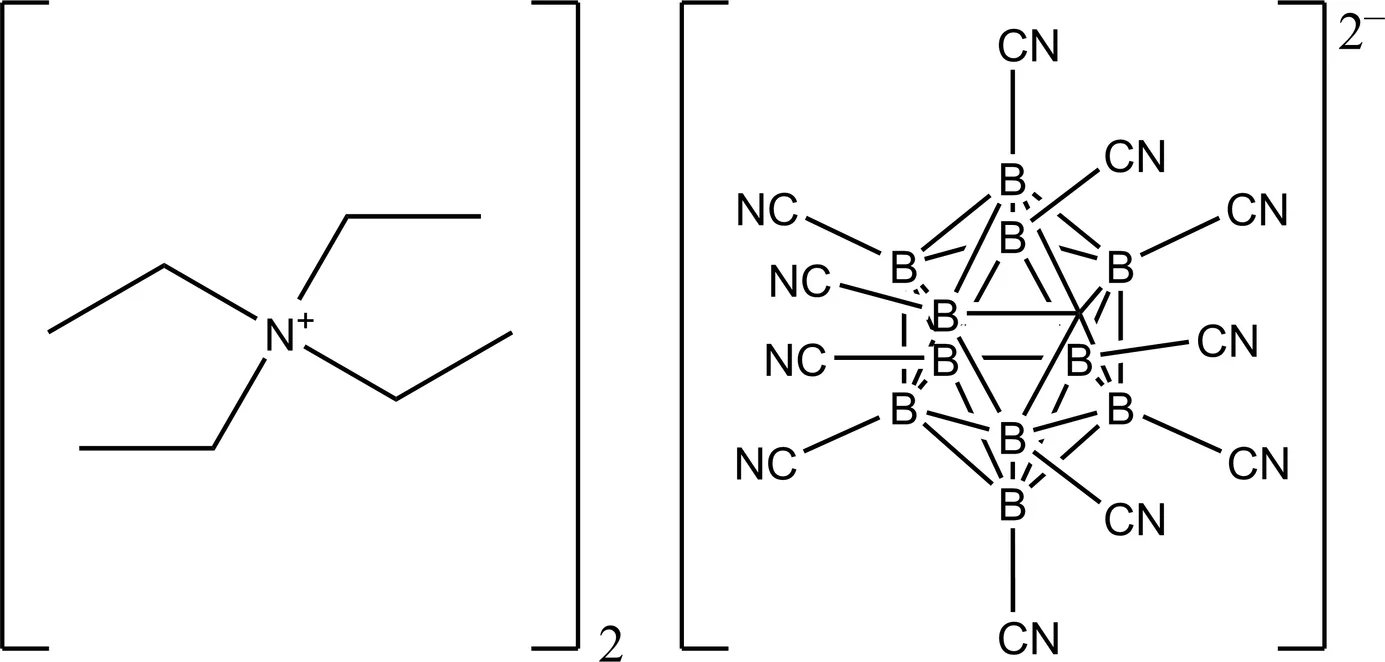


## 2. Structural commentary

**Figure 1:**
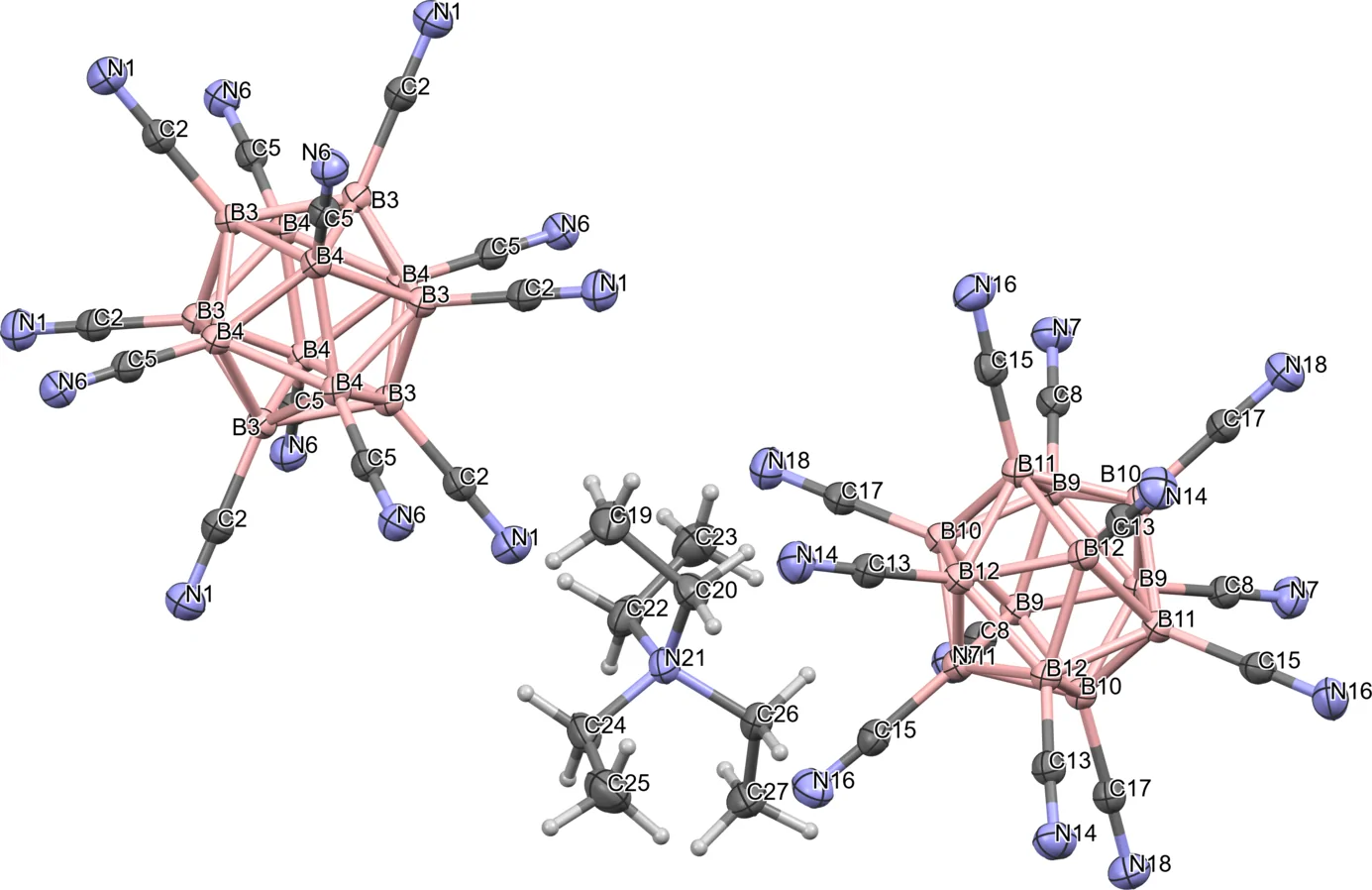
Molecular structure of the title compound with atom labels and ellipsoids at the 50% probability level. Symmetry codes: (i) 1 − *y*, *x* − *y*, *z*; (ii) 1 + *y* − *x*, 1 − *x*, *z* (iii) −*x*, −*y*, −*z*; (iv) −*y* + *x*, *x*, −*z*; (v) −*y*, *x* − *y*, *z*; (vi) *y* − *x*, −*x*, *z*; (vii) *y*, −*x* + *y*, −*z*.

The title salt (Fig. 1 ▸) crystallizes in the trigonal space group *P*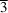 with three 2C_8_H_20_N^+^·B_12_C_12_N_12_^2–^ formula units in the unit cell. The B_12_(CN)_12_^2–^ subunit has nearly icosa­hedral symmetry. B—B bonds within the B12 skeleton range from 1.784 (2) to 1.802 (2) Å; all B—C bond lengths are nearly identical, each falling within 0.007 Å of the average B—C distance of 1.547 Å; the C≡N lengths also fall in a narrow range from 1.111 (2) to 1.147 (2) Å. The B—C≡N angles are all nearly linear, ranging from 179.8 (2) to 173.4 (1)°. The tetra­ethyl­ammonium cation has a tetra­hedral geometry about the central nitro­gen atom and no unusual bond lengths or angles.

## 3. Supra­molecular features

**Figure 2:**
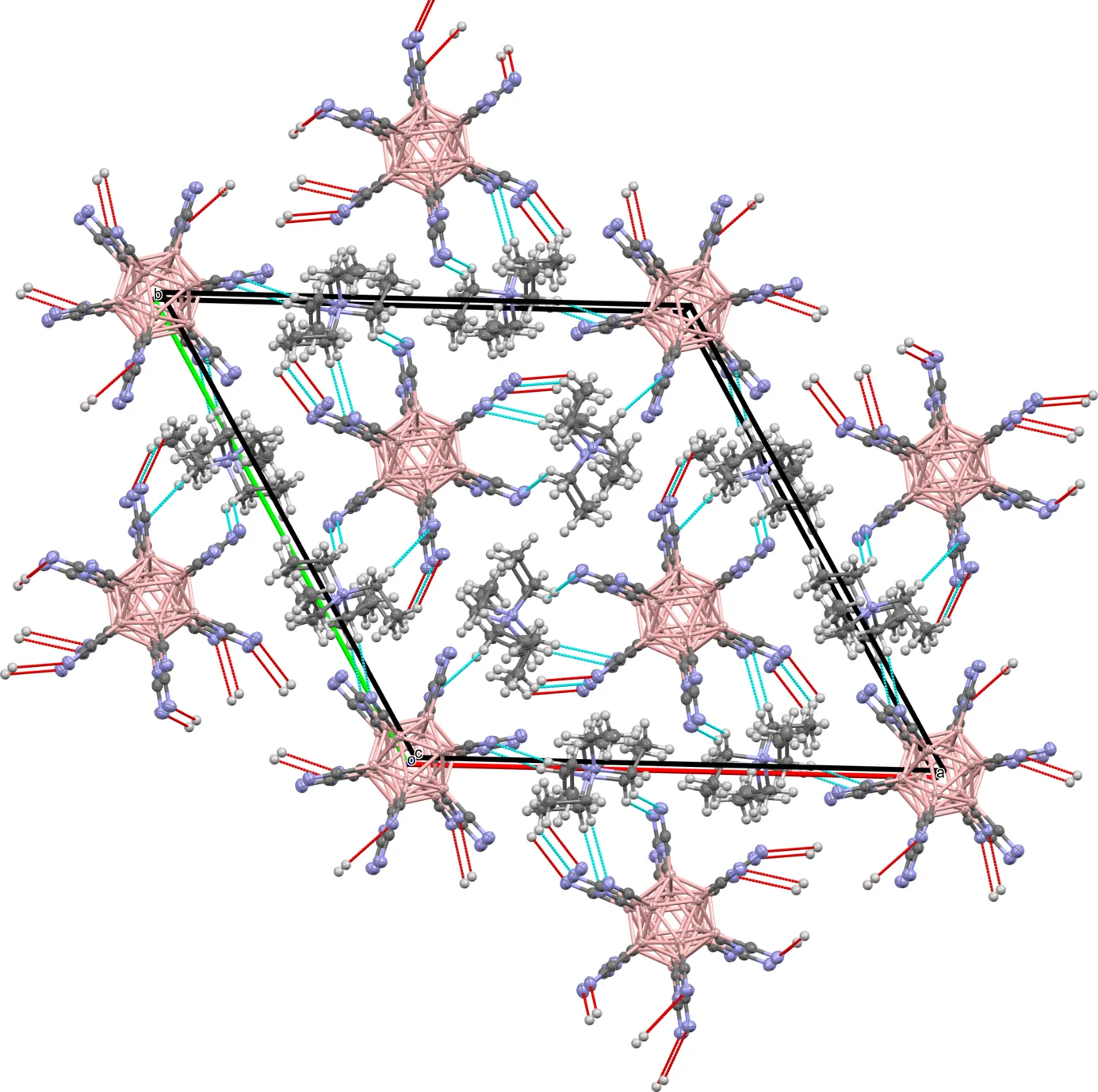
Packing diagram along the *c* crystallographic axis with C—H⋯N hydrogen bonds shown as dashed lines.

Neither the Et_4_N^+^ cation nor the B_12_(CN)_12_^2–^ anion in the title compound contains functional groups capable of conventional hydrogen-bond donation. However, multiple weak C*sp*^3^—H⋯N hydrogen bonds are present, with H⋯N distances shorter than the sum of the van der Waals radii for these atoms (Table 1 ▸, Fig. 2 ▸). The closest cation–anion contact is between N6 in a cyano group in the anion and the methyl­ene hydrogen H22*b* in the cation, with an N6⋯H22*b* distance of 2.496 (1).

## 4. Database survey

A search of the Cambridge Structural Database (Update 6.01 February 2026; Groom *et al.*, 2016 ▸) using ConQuest (version 2025.1 May 2025; Bruno *et al.*, 2002 ▸) revealed only two related structures that contain the dodeca­cyano­dodeca­borate(2−) moiety. One of these structures is a Cu^I^ aceto­nitrile complex [Cu_2_B_12_(CN)_12_(CH_3_CN)_6_] that crystallizes in the *P*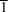 space group and features the B_12_(CN)_12_^2–^ dianion coordinated to each of two Cu^I^ centers *via* a 1.999 Å Cu—N bond (Kamin & Juhasz, 2020 ▸). The other structure (Mayer *et al.*, 2020 ▸) is a tetra­phenyl­phospho­nium salt [(C_6_H_5_)_4_P]_2_[B_12_(CN)_12_] that crystallizes in the *P*2_1_/*n* space group and features a cation–anion contact of 2.725 Å between C**N** and Ph-**H**, just within the carbon/hydrogen van der Waals distance of 2.75 Å.

## 5. Synthesis and crystallization

The title compound was synthesized from the tetra­ethyl­ammonium salt of dodeca­iodo­dodeca­borate (2–) (Juhasz *et al.*, 2016 ▸) by microwave-promoted cyanation in the presence of copper(I) cyanide and palladium(II) chloride in DMF (Kamin & Juhasz, 2020 ▸).

Yield = 35%. ^11^B NMR (acetone-*d*_6_, ppm) for the title compound: δ = −17.6 (*s*); ^1^H NMR (acetone-*d*_6_, ppm) δ = 1.46 [*tt*, 12H, N(CH_2_C**H**_3_)_4_], 3.56 [*q*, 8H, N(C**H**_2_CH_3_)_4_]; ^13^C NMR (acetone-*d*_6_, ppm) δ = 117.6 [*br*, (**C**N)12], 52.6 [N(**C**H_2_CH_3_)_4_] 7.2 [N(CH_2_**C**H_3_)_4_]. FTIR (ATR, cm^−1^) 2986 (*m*), 2954 (*m*), 2924 (*m*), 2235 (*m*), 1704 (*m*), 1616 (*s*). LC-MS *m*/*z* = 220.9. HRMS (for the most abundant mass within each isotopic envelope) *m*/*z* = 221.0784 ([B_12_(CN)_12_]^2–^, theoretical = 221.0777), 572.3154 ([Et_4_N][B_12_(CN)_12_]^−^, theoretical = 572.3160), 443.1622 (H[B_12_(CN)_12_]^−^, theoretical = 443.1638).

Crystals were grown by slow evaporation of a saturated solution of the compound dissolved in 5:1 H_2_O–aceto­nitrile. A suitable crystal for diffraction was selected from the solution and fractured to give an appropriately sized fragment.

## 6. Refinement

Crystal data, data collection and structure refinement details are summarized in Table 2 ▸. Hydrogen atoms were placed at calculated positions and refined as riding with C—H = 0.98 Å for CH_3_ groups and C—H = 0.99 Å for CH_2_ groups and *U*_iso_(H) = 1.2*U*_eq_(C) for CH_2_ groups and *U*_iso_(H) = 1.5*U*_eq_(C) for CH_3_ groups.

## Supplementary Material

Crystal structure: contains datablock(s) I. DOI: 10.1107/S2056989026008480/yy2024sup1.cif

Structure factors: contains datablock(s) I. DOI: 10.1107/S2056989026008480/yy2024Isup2.hkl

CCDC reference: 2051082

Additional supporting information:  crystallographic information; 3D view; checkCIF report

## Figures and Tables

**Table 1:** Hydrogen-bond geometry (Å, °)

*D*—H⋯*A*	*D*—H	H⋯*A*	*D*⋯*A*	*D*—H⋯*A*
C20—H20*b*⋯N14^i^	0.99	2.72 (1)	3.6826 (17)	165 (1)
C22—H22*b*⋯N6	0.99	2.50 (1)	3.4716 (17)	169 (1)
C26—H26*a*⋯N16^ii^	0.99	2.69 (1)	3.4422 (17)	133 (1)
C24—H24*a*⋯N1^iii^	0.99	2.83 (1)	3.6183 (18)	137 (1)
C24—H24*b*⋯N14^iv^	0.99	3.02 (1)	3.6812 (17)	125 (1)
C27—H27*a*⋯N7^v^	0.98	2.75 (1)	3.4681 (18)	131 (1)
C19—H19*b*⋯N18	0.98	2.66 (1)	3.4792 (18)	142 (1)

Symmetry codes: (i) 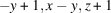; (ii) 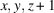; (iii) 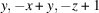; (iv) 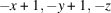; (v) 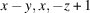.

**Table 2:** Experimental details

Crystal data	
Chemical formula	2C_8_H_20_N^+^·B_12_C_12_N_12_^2−^
*M*_r_	702.62
Crystal system, space group	Trigonal, *P* 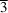
Temperature (K)	101
*a*, *c* (Å)	20.87937 (17), 7.83528 (8)
*V* (Å^3^)	2958.15 (5)
*Z*	3
Radiation type	Cu *K*α
μ (mm^−1^)	0.55
Crystal size (mm)	0.19 × 0.14 × 0.13

Data collection	
Diffractometer	Xcalibur, Onyx, Nova
Absorption correction	Gaussian (*CrysAlis PRO*; Rigaku OD, 2015 ▸)
*T*_min_, *T*_max_	0.798, 1.000
No. of measured, independent and observed [*I* ≥ 2u(*I*)] reflections	36416, 4054, 3537
*R*_int_	0.042
(sin θ/λ)_max_ (Å^−1^)	0.627

Refinement	
*R*[*F*^2^ > 2σ(*F*^2^)], *wR*(*F*^2^), *S*	0.038, 0.107, 1.05
No. of reflections	4054
No. of parameters	268
H-atom treatment	H-atom parameters constrained
Δρ_max_, Δρ_min_ (e Å^−3^)	0.24, −0.31

Computer programs: *CrysAlis PRO* (Rigaku OD, 2015 ▸), *OLEX2* (Dolomanov *et al.*, 2009 ▸; Bourhis *et al.*, 2015 ▸), *Mercury* (Macrae *et al.*, 2020 ▸), *publCIF* (Westrip, 2010 ▸) and *CHEMDRAW 23.1* (Revvity Signals, 2026 ▸).

## supplementary crystallographic information

### Bis(tetraethylammonium) dodecacyanododecaborate(2-) Crystal data

**Table:**

2C_8_H_20_N^+^·B_12_C_12_N_12_^2^^−^	*D*_x_ = 1.183 Mg m^−^^3^
*M**_r_* = 702.62	Cu *K*α radiation, λ = 1.54184 Å
Trigonal, *P*3	Cell parameters from 15853 reflections
*a* = 20.87937 (17) Å	θ = 4.2–75.1°
*c* = 7.83528 (8) Å	µ = 0.55 mm^−^^1^
*V* = 2958.15 (5) Å^3^	*T* = 101 K
*Z* = 3	Prism, clear colourless
*F*(000) = 1101.327	0.19 × 0.14 × 0.13 mm

### Bis(tetraethylammonium) dodecacyanododecaborate(2-) Data collection

**Table:**

Xcalibur, Onyx, Nova diffractometer	3537 reflections with *I*≥ 2u(*I*)
Detector resolution: 8.3552 pixels mm^-1^	*R*_int_ = 0.042
ω scans	θ_max_ = 75.2°, θ_min_ = 2.4°
Absorption correction: gaussian (CrysAlisPro; Rigaku OD, 2015)	*h* = −25→25
*T*_min_ = 0.798, *T*_max_ = 1.000	*k* = −25→25
36416 measured reflections	*l* = −9→9
4054 independent reflections	

### Bis(tetraethylammonium) dodecacyanododecaborate(2-) Refinement

**Table:**

Refinement on *F*^2^	0 restraints
Least-squares matrix: full	8 constraints
*R*[*F*^2^ > 2σ(*F*^2^)] = 0.038	H-atom parameters constrained
*wR*(*F*^2^) = 0.107	*w* = 1/[σ^2^(*F*_o_^2^) + (0.0557*P*)^2^ + 0.7451*P*] where *P* = (*F*_o_^2^ + 2*F*_c_^2^)/3
*S* = 1.05	(Δ/σ)_max_ = 0.001
4054 reflections	Δρ_max_ = 0.24 e Å^−^^3^
268 parameters	Δρ_min_ = −0.31 e Å^−^^3^

### Bis(tetraethylammonium) dodecacyanododecaborate(2-) Fractional atomic coordinates and isotropic or equivalent isotropic displacement parameters (Å^2^)

**Table:**

	*x*	*y*	*z*	*U*_iso_*/*U*_eq_	
N21	0.34564 (5)	0.32269 (6)	0.54461 (14)	0.0279 (2)	
N7	0.60938 (6)	0.17919 (6)	0.48519 (14)	0.0330 (3)	
N18	0.43120 (6)	0.18426 (6)	0.15683 (16)	0.0358 (3)	
N14	0.73019 (6)	0.49910 (6)	−0.34953 (14)	0.0341 (3)	
C15	0.55536 (6)	0.39676 (6)	−0.01584 (15)	0.0262 (3)	
C13	0.71055 (6)	0.44991 (6)	−0.25823 (15)	0.0253 (2)	
N16	0.51540 (7)	0.41678 (6)	−0.03207 (15)	0.0362 (3)	
C17	0.49234 (6)	0.22346 (6)	0.13233 (15)	0.0252 (2)	
C8	0.62552 (6)	0.22195 (6)	0.37884 (15)	0.0243 (2)	
B10	0.57565 (7)	0.27594 (7)	0.09724 (16)	0.0218 (2)	
C20	0.36457 (7)	0.27048 (7)	0.45107 (17)	0.0309 (3)	
H20a	0.39309 (7)	0.29568 (7)	0.34724 (17)	0.035 (4)*	
H20b	0.39698 (7)	0.26050 (7)	0.52524 (17)	0.033 (4)*	
B11	0.60926 (7)	0.36695 (7)	0.01513 (16)	0.0219 (2)	
C22	0.29904 (7)	0.28588 (7)	0.70169 (18)	0.0344 (3)	
H22a	0.28774 (7)	0.32145 (7)	0.75844 (18)	0.038 (4)*	
H22b	0.25153 (7)	0.24316 (7)	0.66448 (18)	0.036 (4)*	
C26	0.41885 (7)	0.38975 (7)	0.59355 (17)	0.0302 (3)	
H26a	0.44704 (7)	0.37295 (7)	0.66394 (17)	0.031 (4)*	
H26b	0.44767 (7)	0.41193 (7)	0.48813 (17)	0.034 (4)*	
C24	0.30016 (7)	0.34448 (8)	0.43163 (19)	0.0360 (3)	
H24a	0.25308 (7)	0.29923 (8)	0.40314 (19)	0.038 (4)*	
H24b	0.28781 (7)	0.37716 (8)	0.49819 (19)	0.045 (4)*	
C27	0.41289 (8)	0.44904 (7)	0.6907 (2)	0.0383 (3)	
H27a	0.3870 (5)	0.4678 (4)	0.6205 (6)	0.049 (5)*	
H27b	0.3852 (5)	0.42814 (16)	0.7966 (7)	0.053 (5)*	
H27c	0.46256 (8)	0.4896 (3)	0.7180 (12)	0.053 (5)*	
B9	0.64580 (7)	0.27693 (7)	0.22968 (16)	0.0212 (3)	
B12	0.68768 (7)	0.39015 (7)	−0.11843 (16)	0.0219 (3)	
C19	0.29855 (8)	0.19728 (8)	0.3992 (2)	0.0407 (3)	
H19a	0.2649 (3)	0.20634 (8)	0.3296 (12)	0.058 (5)*	
H19b	0.31545 (10)	0.1687 (3)	0.3329 (13)	0.052 (5)*	
H19c	0.2726 (4)	0.1694 (3)	0.5016 (2)	0.061 (6)*	
C23	0.33424 (8)	0.25910 (8)	0.8313 (2)	0.0429 (3)	
H23a	0.3440 (6)	0.2223 (5)	0.7782 (5)	0.055 (5)*	
H23b	0.3809 (3)	0.30103 (13)	0.8713 (11)	0.059 (5)*	
H23c	0.3006 (3)	0.2366 (6)	0.9283 (7)	0.058 (5)*	
C25	0.33684 (9)	0.38333 (9)	0.2675 (2)	0.0471 (4)	
H25a	0.3020 (2)	0.3913 (6)	0.1997 (7)	0.066 (6)*	
H25b	0.3806 (4)	0.4311 (3)	0.2936 (2)	0.055 (5)*	
H25c	0.3517 (6)	0.3528 (3)	0.2025 (7)	0.063 (6)*	
C2	−0.03993 (6)	0.12892 (6)	0.92264 (15)	0.0245 (2)	
N1	−0.05419 (6)	0.17352 (6)	0.89631 (15)	0.0347 (3)	
B3	−0.02056 (7)	0.06750 (7)	0.95897 (16)	0.0212 (2)	
B4	0.04206 (7)	0.05478 (7)	0.82630 (16)	0.0217 (2)	
N6	0.12165 (6)	0.15241 (6)	0.58135 (13)	0.0291 (2)	
C5	0.08551 (6)	0.10920 (6)	0.68130 (15)	0.0233 (2)	

### Bis(tetraethylammonium) dodecacyanododecaborate(2-) Atomic displacement parameters (Å^2^)

**Table:**

	*U*^11^	*U*^22^	*U*^33^	*U*^12^	*U*^13^	*U*^23^
N21	0.0195 (5)	0.0248 (5)	0.0403 (6)	0.0117 (4)	−0.0003 (4)	−0.0032 (4)
N7	0.0349 (6)	0.0283 (5)	0.0352 (6)	0.0153 (5)	−0.0008 (4)	0.0043 (5)
N18	0.0243 (6)	0.0325 (6)	0.0472 (7)	0.0116 (5)	0.0018 (5)	−0.0002 (5)
N14	0.0370 (6)	0.0304 (6)	0.0355 (6)	0.0171 (5)	0.0044 (5)	0.0060 (5)
C15	0.0228 (6)	0.0223 (6)	0.0290 (6)	0.0079 (5)	−0.0027 (4)	−0.0023 (4)
C13	0.0229 (5)	0.0237 (6)	0.0295 (6)	0.0117 (5)	0.0006 (4)	−0.0002 (5)
N16	0.0385 (6)	0.0327 (6)	0.0439 (6)	0.0226 (5)	−0.0077 (5)	−0.0074 (5)
C17	0.0244 (6)	0.0223 (5)	0.0303 (6)	0.0128 (5)	−0.0002 (4)	−0.0005 (4)
C8	0.0216 (5)	0.0217 (5)	0.0300 (6)	0.0112 (5)	−0.0012 (4)	−0.0015 (5)
B10	0.0191 (6)	0.0191 (6)	0.0270 (6)	0.0095 (5)	−0.0003 (5)	−0.0004 (5)
C20	0.0258 (6)	0.0298 (6)	0.0419 (7)	0.0175 (5)	0.0017 (5)	−0.0021 (5)
B11	0.0199 (6)	0.0191 (6)	0.0262 (6)	0.0094 (5)	−0.0002 (5)	0.0003 (5)
C22	0.0218 (6)	0.0287 (6)	0.0465 (7)	0.0079 (5)	0.0058 (5)	−0.0041 (5)
C26	0.0194 (6)	0.0253 (6)	0.0418 (7)	0.0081 (5)	−0.0007 (5)	0.0007 (5)
C24	0.0280 (6)	0.0345 (7)	0.0525 (8)	0.0208 (6)	−0.0096 (6)	−0.0088 (6)
C27	0.0318 (7)	0.0248 (6)	0.0554 (8)	0.0119 (6)	−0.0071 (6)	−0.0057 (6)
B9	0.0182 (6)	0.0186 (5)	0.0269 (6)	0.0093 (5)	−0.0001 (4)	−0.0004 (5)
B12	0.0184 (6)	0.0191 (6)	0.0279 (6)	0.0092 (5)	0.0005 (5)	0.0008 (5)
C19	0.0323 (7)	0.0336 (7)	0.0581 (9)	0.0180 (6)	−0.0033 (6)	−0.0134 (6)
C23	0.0374 (8)	0.0364 (7)	0.0457 (8)	0.0116 (6)	0.0070 (6)	0.0062 (6)
C25	0.0515 (9)	0.0491 (9)	0.0512 (9)	0.0329 (8)	−0.0102 (7)	0.0006 (7)
C2	0.0203 (5)	0.0222 (6)	0.0285 (6)	0.0087 (5)	0.0006 (4)	0.0009 (4)
N1	0.0330 (6)	0.0302 (6)	0.0449 (6)	0.0187 (5)	0.0017 (5)	0.0022 (5)
B3	0.0184 (6)	0.0185 (6)	0.0269 (6)	0.0094 (5)	0.0005 (5)	0.0000 (5)
B4	0.0183 (6)	0.0189 (6)	0.0279 (6)	0.0093 (5)	−0.0006 (5)	0.0000 (5)
N6	0.0267 (5)	0.0275 (5)	0.0329 (5)	0.0135 (4)	0.0021 (4)	0.0025 (4)
C5	0.0204 (5)	0.0220 (5)	0.0288 (6)	0.0115 (4)	−0.0015 (4)	−0.0020 (4)

### Bis(tetraethylammonium) dodecacyanododecaborate(2-) Geometric parameters (Å, º)

**Table:**

N21—C20	1.5206 (15)	C24—H24b	0.9900
N21—C22	1.5180 (16)	C24—C25	1.509 (2)
N21—C26	1.5178 (15)	C27—H27a	0.9800
N21—C24	1.5245 (16)	C27—H27b	0.9800
N7—C8	1.1421 (16)	C27—H27c	0.9800
N18—C17	1.1364 (16)	B9—B9^i^	1.786 (2)
N14—C13	1.1462 (16)	B9—B9^ii^	1.786 (2)
C15—N16	1.1118 (17)	B12—B12^i^	1.800 (2)
C15—B11	1.5534 (17)	B12—B12^ii^	1.800 (2)
C13—B12	1.5455 (17)	C19—H19a	0.9800
C17—B10	1.5479 (17)	C19—H19b	0.9800
C8—B9	1.5417 (17)	C19—H19c	0.9800
B10—B11	1.7843 (17)	C23—H23a	0.9800
B10—B11^i^	1.7846 (17)	C23—H23b	0.9800
B10—B9^ii^	1.7877 (18)	C23—H23c	0.9800
B10—B9	1.7867 (18)	C25—H25a	0.9800
B10—B12^i^	1.8018 (18)	C25—H25b	0.9800
C20—H20a	0.9900	C25—H25c	0.9800
C20—H20b	0.9900	C2—N1	1.1292 (16)
C20—C19	1.5146 (18)	C2—B3	1.5519 (17)
B11—B9^ii^	1.7964 (18)	B3—B3^iii^	1.7857 (15)
B11—B12	1.7938 (18)	B3—B3^iv^	1.7857 (15)
B11—B12^i^	1.7928 (18)	B3—B4^v^	1.7888 (18)
C22—H22a	0.9900	B3—B4	1.7910 (18)
C22—H22b	0.9900	B3—B4^iii^	1.7962 (18)
C22—C23	1.515 (2)	B4—B4^vi^	1.796 (2)
C26—H26a	0.9900	B4—B4^v^	1.796 (2)
C26—H26b	0.9900	B4—C5	1.5408 (17)
C26—C27	1.5105 (18)	N6—C5	1.1471 (16)
C24—H24a	0.9900		
			
C22—N21—C20	111.00 (10)	H27c—C27—H27b	109.5
C26—N21—C20	106.27 (9)	B10—B9—C8	120.88 (9)
C26—N21—C22	110.92 (10)	B10^i^—B9—C8	120.82 (9)
C24—N21—C20	110.98 (10)	B11^i^—B9—C8	118.65 (9)
C24—N21—C22	106.54 (10)	B11^i^—B9—B10	59.74 (7)
C24—N21—C26	111.20 (10)	B11^i^—B9—B10^i^	59.72 (7)
B11—C15—N16	177.20 (13)	B9^i^—B9—C8	124.36 (8)
B12—C13—N14	173.43 (13)	B9^ii^—B9—C8	124.42 (8)
B10—C17—N18	179.13 (13)	B9^ii^—B9—B10^i^	107.84 (7)
B9—C8—N7	177.53 (12)	B9^i^—B9—B10^i^	59.99 (8)
B11—B10—C17	121.49 (10)	B9^ii^—B9—B10	60.05 (8)
B11^i^—B10—C17	121.57 (10)	B9^i^—B9—B10	107.89 (7)
B9—B10—C17	121.98 (10)	B9^i^—B9—B11^i^	107.76 (6)
B9^ii^—B10—C17	121.95 (10)	B9^ii^—B9—B11^i^	107.80 (6)
B9—B10—B11^i^	60.40 (7)	B10^ii^—B12—C13	114.83 (9)
B9^ii^—B10—B11^i^	108.25 (9)	B11^ii^—B12—C13	119.11 (9)
B9—B10—B11	108.27 (9)	B11—B12—C13	120.22 (9)
B9^ii^—B10—B11	60.38 (7)	B11^ii^—B12—B10^ii^	59.52 (7)
B12^i^—B10—C17	120.54 (10)	B11—B12—B10^ii^	59.52 (7)
B12^i^—B10—B11^i^	60.02 (7)	B12^ii^—B12—C13	127.28 (8)
B12^i^—B10—B11	59.99 (7)	B12^i^—B12—C13	128.02 (8)
B12^i^—B10—B9	108.77 (9)	B12^i^—B12—B10^ii^	107.48 (6)
B12^i^—B10—B9^ii^	108.73 (9)	B12^ii^—B12—B10^ii^	107.51 (6)
H20a—C20—N21	108.54 (6)	B12^i^—B12—B11	59.86 (8)
H20b—C20—N21	108.54 (6)	B12^ii^—B12—B11^ii^	59.91 (8)
H20b—C20—H20a	107.5	B12^i^—B12—B11^ii^	107.61 (7)
C19—C20—N21	114.90 (10)	B12^ii^—B12—B11	107.56 (7)
C19—C20—H20a	108.54 (8)	H19a—C19—C20	109.5
C19—C20—H20b	108.54 (8)	H19b—C19—C20	109.5
B10—B11—C15	120.33 (10)	H19b—C19—H19a	109.5
B10^ii^—B11—C15	121.93 (10)	H19c—C19—C20	109.5
B9^ii^—B11—C15	119.63 (10)	H19c—C19—H19a	109.5
B9^ii^—B11—B10	59.90 (7)	H19c—C19—H19b	109.5
B9^ii^—B11—B10^ii^	59.86 (7)	H23a—C23—C22	109.5
B12^i^—B11—C15	122.13 (10)	H23b—C23—C22	109.5
B12—B11—C15	123.16 (10)	H23b—C23—H23a	109.5
B12—B11—B10^ii^	60.47 (7)	H23c—C23—C22	109.5
B12^i^—B11—B10^ii^	108.53 (9)	H23c—C23—H23a	109.5
B12—B11—B10	108.53 (9)	H23c—C23—H23b	109.5
B12^i^—B11—B10	60.49 (7)	H25a—C25—C24	109.5
B12—B11—B9^ii^	108.70 (9)	H25b—C25—C24	109.5
B12^i^—B11—B9^ii^	108.75 (9)	H25b—C25—H25a	109.5
H22a—C22—N21	108.50 (6)	H25c—C25—C24	109.5
H22b—C22—N21	108.50 (6)	H25c—C25—H25a	109.5
H22b—C22—H22a	107.5	H25c—C25—H25b	109.5
C23—C22—N21	115.09 (11)	B3—C2—N1	179.85 (12)
C23—C22—H22a	108.50 (8)	B3^iii^—B3—C2	121.29 (9)
C23—C22—H22b	108.50 (8)	B3^iv^—B3—C2	122.00 (9)
H26a—C26—N21	108.47 (6)	B4—B3—C2	121.82 (9)
H26b—C26—N21	108.47 (7)	B4^v^—B3—C2	121.34 (10)
H26b—C26—H26a	107.5	B4^iii^—B3—C2	121.07 (9)
C27—C26—N21	115.19 (10)	B4^v^—B3—B3^iv^	108.39 (6)
C27—C26—H26a	108.47 (7)	B4—B3—B3^iv^	60.29 (6)
C27—C26—H26b	108.47 (8)	B4^iii^—B3—B3^iv^	59.92 (8)
H24a—C24—N21	108.40 (7)	B4^v^—B4—B3^vi^	107.77 (7)
H24b—C24—N21	108.40 (6)	B4^vi^—B4—B3^iv^	107.69 (6)
H24b—C24—H24a	107.5	B4^vi^—B4—B3^vi^	59.95 (8)
C25—C24—N21	115.53 (11)	B4^v^—B4—B3^iv^	107.62 (6)
C25—C24—H24a	108.40 (8)	B4^vi^—B4—B3	107.67 (7)
C25—C24—H24b	108.40 (8)	B4^v^—B4—B3	59.83 (8)
H27a—C27—C26	109.5	C5—B4—B3	121.53 (9)
H27b—C27—C26	109.5	C5—B4—B3^vi^	119.17 (9)
H27b—C27—H27a	109.5	C5—B4—B3^iv^	117.01 (9)
H27c—C27—C26	109.5	C5—B4—B4^v^	126.55 (8)
H27c—C27—H27a	109.5	N6—C5—B4	175.28 (12)
			
C15—B11—B10—C17	2.61 (12)	B11—B12—B11^ii^—B12^ii^	100.81 (12)
C15—B11—B10^ii^—C17^ii^	−3.30 (13)	B11—B12—B12^i^—C13^i^	105.40 (10)
C15—B11—B10—B11^i^	149.86 (12)	B11—B12—B12^ii^—C13^ii^	153.60 (8)
C15—B11—B10^ii^—B11^ii^	−150.51 (12)	B11—B12—B12^ii^—B11^ii^	−99.85 (4)
C15—B11—B10—B9	−146.28 (11)	B11^ii^—B12—B12^i^—B11	99.81 (4)
C15—B11—B10^ii^—B9^ii^	108.16 (14)	B11—B12—B12^ii^—B11^i^	−0.04 (11)
C15—B11—B10^ii^—B9^i^	145.64 (11)	B11—B12—B12^i^—B11^i^	−99.85 (4)
C15—B11—B10—B9^ii^	−108.84 (14)	B11—B12^i^—B12—B12^ii^	137.58 (6)
C15—B11—B10^ii^—B12	−112.81 (14)	B11—B12—B12^i^—B12^ii^	−137.58 (6)
C15—B11—B10—B12^i^	112.15 (14)	B11—B12^i^—B12^ii^—B12	−37.76 (7)
C15—B11—B9^ii^—C8^ii^	−0.92 (12)	B11—B12—B12^ii^—B12^i^	37.73 (7)
C15—B11—B9^ii^—B10^ii^	−111.93 (13)	C22—N21—C20—C19	55.98 (12)
C15—B11—B9^ii^—B10	109.99 (13)	C22—N21—C26—C27	−58.61 (11)
C15—B11—B9^ii^—B9	147.32 (12)	C22—N21—C24—C25	179.24 (11)
C15—B11—B9^ii^—B9^i^	−149.33 (12)	C26—N21—C20—C19	176.67 (11)
C15—B11—B12—C13	8.09 (13)	C26—N21—C22—C23	−59.37 (11)
C15—B11—B12^i^—C13^i^	−5.96 (12)	C26—N21—C24—C25	58.26 (12)
C15—B11—B12—B10^ii^	110.86 (14)	C24—N21—C20—C19	−62.30 (12)
C15—B11—B12^i^—B10	−109.25 (14)	C24—N21—C22—C23	179.46 (10)
C15—B11—B12—B11^ii^	148.16 (10)	C24—N21—C26—C27	59.76 (11)
C15—B11—B12^i^—B11^i^	−146.55 (9)	B9—B10—B11^i^—C15^i^	108.16 (8)
C15—B11—B12^i^—B12	112.64 (13)	B9—B10—B11^i^—B10^i^	−37.36 (8)
C15—B11—B12—B12^i^	−110.99 (13)	B9^ii^—B10—B11^i^—B9	37.48 (9)
C15—B11—B12^i^—B12^ii^	150.44 (10)	B9—B10—B11—B9^ii^	−37.44 (9)
C15—B11—B12—B12^ii^	−148.78 (10)	B9^ii^—B10—B11—B12	101.33 (7)
C13—B12—B10^ii^—C17^ii^	0.61 (11)	B9—B10^i^—B11^ii^—B12	−63.89 (9)
C13—B12—B10^ii^—B11	111.79 (13)	B9^i^—B10^ii^—B11—B12	−101.54 (12)
C13—B12—B10^ii^—B11^ii^	−110.46 (13)	B9—B10—B11^i^—B12^i^	−139.03 (9)
C13—B12—B10^ii^—B9^ii^	148.82 (10)	B9—B10—B11—B12	63.89 (9)
C13—B12—B10^ii^—B9^i^	−147.48 (10)	B9—B10—B11—B12^i^	101.57 (11)
C13—B12—B11^ii^—C15^ii^	−5.96 (12)	B9^ii^—B10—B11—B12^i^	139.02 (9)
C13—B12—B11—B10^ii^	−102.77 (13)	B9—B10—B11^i^—B12^ii^	−101.38 (7)
C13—B12—B11—B10	156.88 (11)	B9—B10—B9^ii^—C8^ii^	−114.48 (7)
C13—B12—B11^ii^—B10^ii^	103.30 (13)	B9—B10—B9^ii^—B10^ii^	100.93 (8)
C13—B12—B11^ii^—B10^i^	−156.35 (10)	B9—B10—B9^ii^—B9^i^	37.58 (9)
C13—B12—B11—B9^ii^	−139.54 (11)	B9^ii^—B10—B9—B9^i^	−37.59 (9)
C13—B12—B11^ii^—B9^i^	140.11 (10)	B9—B10—B12^i^—C13^i^	148.82 (9)
C13—B12—B11^ii^—B12^ii^	−118.60 (14)	B9—B10—B12^i^—B11^i^	37.02 (9)
C13—B12—B11—B12^i^	119.08 (15)	B9—B10^i^—B12^ii^—B12	63.51 (9)
C13—B12—B12^i^—C13^i^	−1.15 (14)	B9^ii^—B10—B12^i^—B12	0.27 (9)
C13—B12—B12^ii^—C13^ii^	−1.15 (14)	B9—B10—B12^i^—B12	−63.43 (9)
C13—B12—B12^i^—B10	−143.66 (13)	B9—B10—B12^i^—B12^ii^	−0.20 (9)
C13—B12—B12^ii^—B10^i^	142.46 (13)	B9—B11^i^—B10—B12^i^	139.03 (9)
C13—B12—B12^i^—B11^i^	153.60 (12)	B9^ii^—B11—B10—B12^i^	−139.02 (9)
C13—B12—B12^i^—B11	−106.55 (17)	B9—B11^i^—B12^ii^—B12	−63.50 (8)
C13—B12—B12^ii^—B11^i^	−154.79 (12)	B9^ii^—B11—B12—B12^i^	101.38 (10)
C13—B12—B12^ii^—B11^ii^	105.40 (17)	B9—B11^i^—B12^i^—B12	63.59 (9)
C13—B12—B12^ii^—B12^i^	−117.03 (11)	B9^ii^—B11—B12^i^—B12	−101.30 (10)
C13—B12—B12^i^—B12^ii^	115.87 (11)	B9—B9^i^—B10^ii^—B12	−63.75 (7)
C17—B10—B11^i^—C15^i^	−3.30 (13)	B9^ii^—B9—B10—B12^i^	101.26 (7)
C17—B10—B11^i^—B10^i^	−148.82 (12)	B9—B9^ii^—B10^ii^—B12	63.67 (7)
C17—B10—B11—B10^ii^	148.80 (12)	B9—B9^ii^—B10—B12^i^	−101.33 (7)
C17—B10—B11—B9^ii^	111.45 (14)	B9—B9^ii^—B11—B10^ii^	−100.75 (12)
C17—B10—B11^i^—B9	−111.46 (14)	B9—B9^ii^—B11—B12	−63.72 (10)
C17—B10—B11—B12^i^	−109.53 (14)	B9^i^—B9^ii^—B11—B12	−0.37 (10)
C17—B10—B11^i^—B12^ii^	147.16 (11)	B9—B9^i^—B11^ii^—B12	63.61 (10)
C17—B10—B11—B12	−147.22 (11)	B9—B9^ii^—B11—B12^i^	0.26 (10)
C17—B10—B11^i^—B12^i^	109.52 (14)	B12—B10^ii^—B11—B9^ii^	139.03 (9)
C17—B10—B9—C8	3.47 (12)	B12—B10^ii^—B11—B12^i^	37.65 (8)
C17—B10—B9^ii^—C8^ii^	−3.36 (13)	B12^i^—B10—B11—B12	−37.69 (8)
C17—B10—B9—B10^i^	148.02 (9)	B12—B11—B10^ii^—C17^ii^	109.52 (8)
C17—B10—B9^ii^—B10^ii^	−147.94 (9)	B12—B11—B10^ii^—B11^ii^	−37.70 (8)
C17—B10—B9—B11^i^	110.80 (14)	B12—B11—B10^ii^—B9^ii^	−139.03 (9)
C17—B10—B9^ii^—B11	−110.71 (14)	B12—B11—B9^ii^—C8^ii^	148.04 (9)
C17—B10—B9—B9^i^	−148.67 (10)	B12—B11—B9^ii^—B10^ii^	37.03 (9)
C17—B10—B9—B9^ii^	−111.08 (13)	B12—B11—B12^i^—C13^i^	−118.60 (7)
C17—B10—B9^ii^—B9^i^	148.70 (10)	B12—B11—B12^i^—B11^i^	100.81 (8)
C17—B10—B9^ii^—B9	111.12 (13)	B12^i^—B11—B12—B12^ii^	−37.79 (9)
C17—B10—B12^i^—C13^i^	0.61 (12)	B12—B11—B12^i^—B12^ii^	37.80 (9)
C17—B10—B12^i^—B11^i^	−111.18 (14)	B12—B12^i^—B10—B11^i^	−100.45 (11)
C17—B10—B12^i^—B11	111.08 (14)	C2—B3—B3^iii^—C2^iii^	0.79 (10)
C17—B10—B12^i^—B12	148.36 (12)	C2—B3—B3^iv^—C2^iv^	−0.79 (10)
C17—B10—B12^i^—B12^ii^	−148.40 (12)	C2—B3—B3^iv^—B3^vi^	−148.58 (15)
C8—B9—B10^i^—C17^i^	−3.36 (12)	C2—B3—B3^iii^—B3^v^	148.31 (15)
C8—B9—B10—B11^i^	−107.33 (14)	C2—B3—B3^iii^—B4^vii^	−147.87 (13)
C8—B9—B10^i^—B11^ii^	−152.16 (11)	C2—B3—B3^iii^—B4^iii^	−110.27 (15)
C8—B9—B10^i^—B11^i^	107.35 (13)	C2—B3—B3^iv^—B4	−111.06 (16)
C8—B9—B10—B11	152.18 (11)	C2—B3—B3^iii^—B4^v^	110.75 (17)
C8—B9—B10^i^—B9^i^	−114.48 (14)	C2—B3—B3^iv^—B4^iii^	109.96 (15)
C8—B9—B10—B9^ii^	114.55 (14)	C2—B3—B3^iv^—B4^iv^	147.64 (13)
C8—B9—B10^i^—B12^ii^	144.19 (10)	C2—B3—B4—B3^iv^	111.34 (14)
C8—B9—B10—B12^i^	−144.19 (11)	C2—B3—B4^iii^—B3^iv^	−111.46 (12)
C8—B9—B11^i^—C15^i^	−0.92 (12)	C2—B3—B4^v^—B3^iii^	−110.66 (14)
C8—B9—B11^i^—B10^i^	−110.91 (13)	C2—B3—B4^v^—B3^v^	−148.00 (9)
C8—B9—B11^i^—B10	111.00 (13)	C2—B3—B4^iii^—B3^iii^	110.62 (12)
C8—B9—B11^i^—B12^ii^	−147.98 (10)	C2—B3—B4—B3^vi^	148.70 (9)
C8—B9—B11^i^—B12^i^	148.04 (10)	C2—B3—B4^v^—B4^vi^	148.91 (9)
C8—B9—B9^ii^—C8^ii^	−0.09 (12)	C2—B3—B4—B4^v^	−110.49 (13)
C8—B9—B9^i^—C8^i^	−0.09 (13)	C2—B3—B4^iii^—B4^iv^	−148.85 (12)
C8—B9—B9^i^—B10^i^	108.77 (16)	C2—B3—B4^iii^—B4^vii^	147.85 (12)
C8—B9—B9^ii^—B10	−108.86 (17)	C2—B3—B4^v^—B4	111.28 (13)
C8—B9—B9^ii^—B10^ii^	150.84 (12)	C2—B3—B4—B4^vi^	−148.10 (10)
C8—B9—B9^i^—B10^ii^	−150.90 (12)	C2—B3—B4^v^—C5^v^	−4.60 (12)
C8—B9—B9^i^—B11^ii^	146.04 (12)	C2—B3—B4^iii^—C5^iii^	−1.83 (12)
C8—B9—B9^ii^—B11	−146.07 (12)	C2—B3—B4—C5	6.34 (13)
C8—B9—B9^ii^—B9^i^	113.23 (11)	B3—B3^iii^—B3^v^—B3^vii^	63.90 (6)
C8—B9—B9^i^—B9^ii^	−113.32 (11)	B3—B3^iv^—B3^vi^—B3^vii^	−63.90 (6)
B10—B11—B10^ii^—C17^ii^	−148.82 (10)	B3—B3^iv^—B3^vi^—B4	37.56 (11)
B10—B11—B10^ii^—B11^ii^	63.97 (7)	B3^vi^—B3^iv^—B3—B4	−37.52 (11)
B10^ii^—B11—B10—B11^i^	−63.96 (7)	B3^iii^—B3—B3^iv^—B4	101.42 (8)
B10—B11^i^—B10^i^—B9	37.35 (9)	B3^v^—B3^iii^—B3—B4	−0.12 (10)
B10—B11—B10^ii^—B9^i^	0.12 (9)	B3—B3^iii^—B3^v^—B4^vi^	0.07 (10)
B10^ii^—B11—B10—B9	−0.10 (9)	B3—B3^iii^—B3^v^—B4^vii^	101.46 (8)
B10—B11—B10^ii^—B9^ii^	−37.36 (9)	B3^vii^—B3^vi^—B4—B3	63.10 (11)
B10^ii^—B11—B10—B12^i^	−101.67 (11)	B3—B3^iii^—B4^vii^—B3^v^	−100.44 (12)
B10—B11—B10^ii^—B12	101.66 (11)	B3^iv^—B3^vi^—B4—B3	−37.34 (10)
B10—B11—B9^ii^—C8^ii^	−110.91 (8)	B3^iv^—B3—B4—B3^vi^	37.36 (10)
B10—B11—B9^ii^—B10^ii^	138.08 (9)	B3^iii^—B3—B4—B3^iv^	−100.44 (12)
B10—B11^i^—B9—B10^i^	−138.08 (9)	B3^vi^—B3^iv^—B4—B3	137.92 (9)
B10—B11^i^—B9—B9^i^	−100.75 (9)	B3^iii^—B3—B4—B3^vi^	−63.08 (11)
B10—B11—B9^ii^—B9^i^	100.67 (9)	B3—B3^iv^—B4—B3^vi^	−137.92 (9)
B10—B11—B9^ii^—B9	37.33 (9)	B3^iv^—B3—B4—B4^v^	138.17 (8)
B10—B11^i^—B9—B9^ii^	−37.41 (9)	B3—B3^iii^—B4^vii^—B4^iv^	−0.01 (10)
B10—B11—B12^i^—C13^i^	103.30 (8)	B3^iii^—B3—B4—B4^vi^	0.12 (10)
B10^ii^—B11—B12^i^—B10	100.36 (10)	B3^iv^—B3—B4—B4^vi^	100.56 (6)
B10—B11—B12—B10^ii^	−100.35 (10)	B3^iii^—B3—B4—B4^v^	37.73 (9)
B10—B11—B12^i^—B11^i^	−37.30 (9)	B3—B3^iv^—B4^iii^—B4^vii^	100.43 (6)
B10^i^—B11^ii^—B12—B11	63.06 (10)	B3—B3^iii^—B4^v^—B4^vi^	100.69 (9)
B10—B11^i^—B12^i^—B11	37.30 (9)	B3—B3^iv^—B4—B4^vi^	−100.52 (9)
B10—B11—B12—B11^ii^	−63.05 (10)	B3^iv^—B3—B4^v^—B4	−37.62 (9)
B10^ii^—B11—B12^i^—B12	−37.75 (8)	B3—B3^iii^—B4^v^—B4	37.39 (9)
B10^ii^—B11—B12—B12^i^	138.15 (9)	B3^iii^—B3—B4^v^—B4	−138.06 (8)
B10—B11—B12^i^—B12	−138.11 (9)	B3—B3^iv^—B4—B4^v^	−37.23 (9)
B10—B11^i^—B12^i^—B12	100.36 (6)	B3^vii^—B3^vi^—B4—C5	−153.50 (9)
B10—B11^i^—B12^ii^—B12	0.05 (9)	B3^iv^—B3^vi^—B4—C5	106.06 (7)
B10—B11—B12^i^—B12^ii^	−100.31 (6)	B3^iii^—B3—B4—C5	154.56 (9)
B10—B11—B12—B12^i^	37.80 (8)	B3^iv^—B3—B4—C5	−105.00 (7)
B10—B11—B12—B12^ii^	0.01 (9)	B3^vi^—B3^iv^—B4—C5	−109.63 (6)
B10—B9—B10^i^—C17^i^	−147.94 (7)	B3—B3^iv^—B4—C5	112.45 (6)
B10—B9—B10^i^—B11^i^	−37.24 (10)	B3—B4—B3^vi^—C2^vi^	−148.00 (7)
B10^i^—B9—B10—B11	−63.27 (9)	B3—B4—B3^iv^—C2^iv^	−110.62 (6)
B10—B9^ii^—B10^ii^—B11	37.22 (10)	B3—B4^iii^—B3^iv^—B3^vi^	−100.44 (6)
B10—B9—B10^i^—B11^ii^	63.26 (9)	B3^iv^—B4^iii^—B3—B4	36.97 (7)
B10^i^—B9—B10—B9^ii^	−100.90 (12)	B3^iv^—B4—B3—B4^iii^	−36.81 (7)
B10—B9—B10^i^—B9^i^	100.93 (12)	B3^iii^—B4^v^—B3—B4	138.06 (11)
B10—B9^ii^—B10^ii^—B12	0.36 (9)	B3^iv^—B4—B3—B4^v^	−138.17 (11)
B10—B9—B10^i^—B12^ii^	−0.39 (9)	B3—B4—B3^iv^—B4^iv^	100.94 (9)
B10—B9—B11^i^—C15^i^	−111.93 (8)	B3—B4^iii^—B3^iv^—B4	−36.87 (7)
B10—B9—B11^i^—B10^i^	138.08 (9)	B3^vi^—B4—B3—B4^v^	−100.81 (12)
B10—B9^ii^—B11—B10^ii^	−138.08 (9)	B3^vi^—B4—B3—B4^iii^	0.55 (8)
B10—B9^ii^—B11—B12	−101.05 (7)	B3—B4—B3^vi^—B4^iv^	−0.47 (8)
B10—B9—B11^i^—B12^i^	37.03 (9)	B3—B4—B3^vi^—B4^vi^	100.72 (12)
B10—B9—B11^i^—B12^ii^	101.02 (7)	B3—B4—B3^iv^—B4^iii^	37.01 (7)
B10—B9^ii^—B11—B12^i^	−37.06 (9)	B3—B4^v^—B3^iii^—B4^vii^	−100.90 (9)
B10—B9—B9^ii^—C8^ii^	108.77 (9)	B3—B4—B4^vi^—B3^v^	−0.08 (11)
B10—B9—B9^i^—C8^i^	150.84 (8)	B3—B4^v^—B4^vi^—B3^vii^	62.92 (6)
B10—B9—B9^i^—B10^ii^	0.03 (10)	B3—B4^v^—B4—B3^iv^	37.17 (9)
B10—B9^ii^—B9—B10^i^	100.33 (4)	B3—B4^iii^—B4^vii^—B3^v^	0.13 (7)
B10—B9—B9^i^—B10^i^	−100.30 (4)	B3—B4—B4^v^—B3^iii^	−37.30 (9)
B10—B9—B9^ii^—B10^ii^	−100.30 (4)	B3—B4^iii^—B4^iv^—B3^vii^	−62.92 (8)
B10—B9—B9^ii^—B11	−37.21 (8)	B3—B4—B4^vi^—B3^vii^	−63.12 (6)
B10—B9^ii^—B9—B11^i^	37.27 (8)	B3—B4^iii^—B4^iv^—B3^vi^	0.13 (7)
B10—B9—B9^i^—B11^ii^	−63.02 (8)	B3—B4—B4^vi^—B3^vi^	−100.30 (4)
B10^i^—B9—B9^ii^—B11	63.12 (8)	B3—B4^v^—B4—B3^vi^	100.22 (4)
B10—B9—B9^i^—B9^ii^	37.61 (7)	B3—B4—B4^v^—B3^v^	−100.30 (4)
B10—B9^ii^—B9—B9^i^	137.91 (6)	B3—B4—B4^vi^—B4^v^	37.53 (7)
B10—B9^ii^—B9^i^—B9	−37.58 (7)	B3—B4^v^—B4—B4^vi^	137.83 (6)
B10—B9—B9^ii^—B9^i^	−137.91 (6)	B3—B4^iii^—B4^vii^—B4^iv^	100.65 (7)
B10—B12^i^—B11^i^—B9	−36.77 (9)	B3—B4^v^—B4^vi^—B4	−37.61 (7)
B10—B12^i^—B11—B9^ii^	36.81 (9)	B3—B4—B4^v^—B4^vi^	−137.83 (6)
B10—B12^i^—B11—B12	138.11 (10)	B3—B4^iii^—B4^iv^—B4^vii^	−100.53 (7)
B10^ii^—B12—B11—B12^i^	−138.15 (10)	B3^iii^—B4^v^—B4—C5	−146.08 (9)
B10^ii^—B12—B12^i^—B10	−0.05 (7)	B3^vii^—B4^vi^—B4—C5	143.56 (9)
B10^i^—B12^ii^—B12—B11	−62.78 (9)	B3—B4—B4^vi^—C5^vi^	150.92 (8)
B10—B12^i^—B12—B11	−37.12 (8)	B3—B4^v^—B4—C5	−108.78 (9)
B10^ii^—B12—B12^i^—B11	37.07 (8)	B3—B4—B4^v^—C5^v^	106.39 (9)
B10—B12^i^—B12—B11^ii^	62.70 (9)	B3^v^—B4^vi^—B4—C5	−153.40 (8)
B10—B12^i^—B12^ii^—B12	−100.51 (7)	B4—B3—B3^iv^—C2^iv^	110.27 (8)
B10—B12^i^—B12—B12^ii^	100.46 (7)	B4—B3—B3^iii^—C2^iii^	−147.64 (9)
C20—N21—C22—C23	58.53 (11)	B4—B3^vi^—B3^vii^—B3^v^	0.07 (7)
C20—N21—C26—C27	−179.36 (10)	B4—B3^iv^—B3^vi^—B3^vii^	−101.46 (8)
C20—N21—C24—C25	−59.82 (11)	B4—B3^iv^—B3—B4^iii^	138.98 (6)
B11—B10—B11^i^—C15^i^	−150.51 (10)	B4—B3—B3^iv^—B4^iv^	−101.30 (6)
B11—B10—B11^i^—B10^i^	63.97 (7)	B4—B3^vi^—B3^iv^—B4^iii^	101.39 (6)
B11—B10—B11^i^—B9	101.33 (11)	B4—B3—B3^iv^—B4^iii^	−138.98 (7)
B11^i^—B10—B11—B9^ii^	−101.31 (11)	B4—B3—B3^iii^—B4^v^	−37.68 (8)
B11—B10^ii^—B11^ii^—B12	37.71 (9)	B4^v^—B3—B3^iv^—B4	37.59 (8)
B11—B10—B11^i^—B12^i^	−37.70 (9)	B4—B3^vi^—B3^vii^—B4^iv^	−101.39 (10)
B11^i^—B10—B11—B12	0.02 (9)	B4—B3—B3^iii^—B4^iii^	101.30 (10)
B11—B10—B11^i^—B12^ii^	−0.05 (9)	B4—B3—B3^iii^—B4^vii^	63.71 (8)
B11—B10—B9^ii^—C8^ii^	107.35 (8)	B4—B3—B4^v^—B3^v^	100.72 (8)
B11—B10—B9^ii^—B10^ii^	−37.24 (9)	B4—B3^vi^—B4^iv^—B3^vii^	100.90 (15)
B11—B10—B9—B11^i^	−100.49 (10)	B4—B3—B4^iii^—B3^iii^	−100.94 (15)
B11^i^—B10—B9^ii^—B11	100.50 (10)	B4^v^—B3—B4—B4^vi^	−37.61 (9)
B11—B10—B9—B9^ii^	37.63 (8)	B4—B3^vi^—B4^iv^—B4^iii^	0.21 (10)
B11^i^—B10—B9—B9^ii^	138.12 (9)	B4—B3^vi^—B4^iv^—B4^vii^	63.51 (9)
B11—B10—B9^ii^—B9	−138.17 (9)	B4—B3—B4^v^—B4^vi^	37.63 (9)
B11—B10—B9^ii^—B9^i^	−100.59 (6)	B4—B3—B4^iii^—B4^iv^	−0.42 (10)
B11—B10—B9—B9^i^	0.04 (9)	B4^iii^—B3—B4^v^—B4	−101.19 (8)
B11^i^—B10—B9^ii^—B9	−37.68 (8)	B4—B3^iv^—B4^iii^—B4^vii^	63.56 (7)
B11—B10^ii^—B9^i^—B9	−0.10 (9)	B4—B3—B4^iii^—B4^vii^	−63.72 (9)
B11—B10^ii^—B9^ii^—B9	100.53 (6)	B4^iii^—B3—B4—B4^v^	101.36 (8)
B11—B10—B12^i^—C13^i^	−110.46 (8)	B4^iii^—B3—B4—B4^vi^	63.75 (7)
B11—B10—B12^i^—B11^i^	137.74 (9)	B4^iii^—B3^iv^—B4—C5	149.47 (9)
B11—B10^ii^—B12—B11^ii^	−137.74 (9)	B4—B3—B4^iii^—C5^iii^	146.60 (9)
B11—B10—B12^i^—B12	37.29 (9)	B4^v^—B3—B4—C5	116.84 (7)
B11—B10—B12^i^—B12^ii^	100.52 (9)	B4—B3—B4^v^—C5^v^	−115.88 (7)
B11—B10^ii^—B12—B12^i^	−37.22 (9)	B4^iii^—B3—B4—C5	−141.81 (10)
B11—B10^ii^—B12—B12^ii^	−100.45 (9)	B4^iv^—B3^vi^—B4—C5	142.93 (9)
B11—B9^ii^—B10—B9	138.17 (10)	B4—B4^vi^—B3^v^—B3^vii^	−100.43 (9)
B11^i^—B9—B10—B9^ii^	−138.12 (10)	B4—B4^vi^—B3^v^—B3^iii^	0.01 (8)
B11—B9^ii^—B10^ii^—B12	−36.86 (9)	B4—B4^vi^—B3^vii^—B3^v^	100.69 (10)
B11—B9^ii^—B10—B12^i^	36.84 (9)	B4—B4^v^—B3^iii^—B4^vii^	−63.51 (10)
B11—B9^ii^—B9—B11^i^	0.07 (8)	B4—B4^vi^—B3^v^—B4^vii^	−63.56 (4)
B11—B9^ii^—B9—B9^i^	100.70 (7)	B4—B4^vi^—B3^vii^—B4^iv^	−0.21 (10)
B11—B9^ii^—B9^i^—B9	−100.63 (7)	B4—B4^vi^—B4^v^—B3^iii^	−100.65 (5)
B11—B12—B10^ii^—C17^ii^	−111.18 (8)	B4—B4^v^—B4^vi^—B3^vii^	100.53 (5)
B11—B12^i^—B10—B11^i^	−137.74 (9)	B4^v^—B4^vi^—B4—C5	−115.79 (6)
B11—B12—B10^ii^—B11^ii^	137.74 (9)	B4^vi^—B4^v^—B4—C5	113.39 (6)
B11—B12—B10^ii^—B9^i^	100.73 (7)	C5—B4—B3^vi^—C2^vi^	−4.60 (12)
B11—B12—B10^ii^—B9^ii^	37.02 (9)	C5—B4—B3^iv^—C2^iv^	1.83 (12)
B11—B12^i^—B10—B9	−100.72 (7)	C5—B4—B3^iv^—B4^iv^	−146.60 (9)
B11—B12^i^—B10—B9^ii^	−37.01 (9)	C5—B4—B3^vi^—B4^vi^	−115.88 (14)
B11—B12—B11^ii^—C15^ii^	−146.55 (7)	C5—B4—B4^v^—B3^v^	150.92 (12)
B11—B12—B11^ii^—B10^ii^	−37.30 (10)	C5—B4—B4^vi^—B3^vi^	106.39 (17)
B11—B12—B11^ii^—B9^i^	−0.49 (9)	C5—B4—B4^v^—C5^v^	−2.40 (13)
B11—B12^i^—B11^i^—B9	0.53 (9)	C5—B4—B4^vi^—C5^vi^	−2.40 (13)
B11^ii^—B12—B11—B12^i^	−100.85 (12)		

Symmetry codes: (i) −*y*+1, *x*−*y*, *z*; (ii) −*x*+*y*+1, −*x*+1, *z*; (iii) *x*−*y*, *x*, −*z*+2; (iv) *y*, −*x*+*y*, −*z*+2; (v) −*y*, *x*−*y*, *z*; (vi) −*x*+*y*, −*x*, *z*; (vii) −*x*, −*y*, −*z*+2.

### Bis(tetraethylammonium) dodecacyanododecaborate(2-) Hydrogen-bond geometry (Å, º)

**Table:**

*D*—H···*A*	*D*—H	H···*A*	*D*···*A*	*D*—H···*A*
C20—H20*b*···N14^viii^	0.99	2.72 (1)	3.6826 (17)	165 (1)
C22—H22*b*···N6	0.99	2.50 (1)	3.4716 (17)	169 (1)
C26—H26*a*···N16^ix^	0.99	2.69 (1)	3.4422 (17)	133 (1)
C24—H24*a*···N1^x^	0.99	2.83 (1)	3.6183 (18)	137 (1)
C24—H24*b*···N14^xi^	0.99	3.02 (1)	3.6812 (17)	125 (1)
C27—H27*a*···N7^xii^	0.98	2.75 (1)	3.4681 (18)	131 (1)
C19—H19*b*···N18	0.98	2.66 (1)	3.4792 (18)	142 (1)

Symmetry codes: (viii) −*y*+1, *x*−*y*, *z*+1; (ix) *x*, *y*, *z*+1; (x) *y*, −*x*+*y*, −*z*+1; (xi) −*x*+1, −*y*+1, −*z*; (xii) *x*−*y*, *x*, −*z*+1.
